# Development of Xanthyletin-Loaded Nanoparticles for the Control of *Leucoagaricus gongylophorus*

**DOI:** 10.3390/molecules30112469

**Published:** 2025-06-05

**Authors:** Cristiane de Melo Cazal, Moacir Rossi Forim, Ana Paula Terezan, Andreia Pereira Matos, Gracielle Oliveira Sabbag Cunha, Maria Fátima das Graças Fernandes da Silva, Paulo Cezar Vieira, Fernando Carlos Pagnocca, João Batista Fernandes

**Affiliations:** 1 Federal Institute of Education, Science and Technology of Southeastern Minas Gerais—Campus Barbacena, Barbacena 36205018, Brazil; 2 Department of Chemistry, Federal University of São Carlos, São Carlos 13565905, Brazil; mrforim@ufscar.br (M.R.F.); apterezanpn@gmail.com (A.P.T.); apereiramatos@ufscar.br (A.P.M.); dmfs@ufscar.br (M.F.d.G.F.d.S.); pcvieira@fcfrp.usp.br (P.C.V.); djbf@ufscar.br (J.B.F.); 3 Federal Institute of Education, Science and Technology of Goiás—Campus Anápolis, Anápolis 75131457, Brazil; gracielle.oliveira@ifg.edu.br; 4 Center for the Study of Social Insects, São Paulo State University Júlio de Mesquita Filho—Campus Rio Claro, Rio Claro 13506900, Brazil; pagnocca@rc.unesp.br

**Keywords:** biocontrol, biopolymers, nanoencapsulation, nanotechnology, symbiotic fungus

## Abstract

This study describes the development, characterization and in vitro evaluation of poly(ε-caprolactone) (PCL) nanoparticles loaded with xanthyletin for the control of *Atta sexdens rubropilosa* through the inhibition of its symbiotic fungus *Leucoagaricus gongylophorus*. Nanoparticles were prepared via interfacial polymer deposition, with formulation NC5 selected based on optimal physicochemical properties. NC5 exhibited an encapsulation efficiency of 98.0%, average particle size of 304 nm and zeta potential of −29.3 mV. Scanning electron microscopy confirmed spherical morphology and the absence of crystalline residues. The formulation remained physically stable for four months at 4 °C. In vitro release showed biphasic behavior, with an initial burst followed by sustained release. Under UV exposure, NC5 enhanced xanthyletin photostability by 15.4-fold compared to the free compound. Fungicidal assays revealed 76% inhibition of fungal growth with NC5, compared to 85% with free xanthyletin. These results support the potential application of xanthyletin-loaded PCL nanoparticles as a stable and controlled delivery system for the biological control of leaf-cutting ants by targeting their fungal mutualist. Further in vivo studies are recommended to assess efficacy under field conditions.

## 1. Introduction

Brazil is a country of continental dimensions that plays a prominent role in global agricultural production. However, agricultural practices are often associated with the extensive use of pest and disease control inputs. Although pesticide use in agricultural pest control is common and has contributed to increased agricultural production, it has also been linked to several significant health and environmental issues, particularly considerable toxicity to beneficial insects, animals and humans [1,2,3,4,5,6]. Despite these concerns, agricultural production would be seriously compromised without pesticides.

More rational and sustainable solutions for food production are currently being explored. A promising market for the development of bioinsecticides and natural insecticides has emerged, as their potential use may not cause environmental damage. This is likely because plants have their own defenses against other plants, viruses, bacteria, fungi, insects and other organisms. These defenses are chemical in nature and generally involve secondary metabolites that can be isolated and used as natural insecticides [7,8,9,10]. Several different natural insecticides have been used or are currently in use, including pyrethroids, rotenoids, veratrum alkaloids, azadirachtin, among others [11,12,13]. Xanthyletin, a coumarin found in most citrus fruits, has been identified as a growth inhibitor of the symbiotic fungus *Leucoagaricus gongylophorus* at a concentration of 25 μg·mL^−1^ [14], in addition to exhibiting fungicidal activity against other fungi [15]. The *L. gongylophorus* fungus lives in symbiosis with leaf-cutting ants *Acromyrmex octospinosus* (Hymenoptera: Formicidae) [16] and *Atta sexdens* (Hymenoptera: Formicidae: Myrmicinae) [17], thus constituting a target for the development of alternative methods to control pest insects, as eliminating this fungus is known to cause the death of these ants [18]. Leaf-cutting ants are responsible for significant losses in coffee, sugarcane, pasture and especially in Eucalyptus, Pinus and reforestation areas [19,20,21]. All species of *Atta* (locally known as saúvas) and *Acromyrmex* (quenquéns), along with certain species from the *Trachymyrmex*, *Sericomyrmex* and *Apterostigma* genera, are classified as leaf-cutting ants [22].

Despite offering several advantages, such as selectivity, low toxicity and biodegradability, the main challenge with using natural products in large-scale agriculture is their low environmental stability under sunlight, temperature fluctuations, microorganisms or even water (hydrolysis). To ensure greater stability, the process of nanoencapsulation serves as an attractive tool for protecting active ingredients against damage and degradation caused by oxidative reactions, light and heat. Furthermore, encapsulation improves solubility in aqueous media, simplifying formulations and application methods, reducing the number of applications by controlling the release of active ingredients, lowering the concentrations of applied dosages and enhancing insecticidal activity compared to the free forms of the active ingredients. It also prevents ants from recognizing the presence of insecticides or fungicides in the formulation [23,24,25,26,27,28,29].

Polymeric nanoparticles can be classified as either nanocapsules (NCs) or nanospheres (NSs). NCs consist of a polymeric wall surrounding an oil core (vesicle), with the active ingredient dissolved within the core and/or adsorbed onto the polymeric wall [30]. NSs consist of a rigid system formed from the homogeneous dispersion of the active compound in a polymer matrix [31,32]. Thus, the present study aimed to prepare and characterize biopolymeric nanoparticles loaded with xanthyletin, both in colloidal dispersions, and to evaluate their potential as growth inhibitors of the symbiotic fungus *L. gongylophorus*, which lives in symbiosis with the leaf-cutting ant *Atta sexdens rubropilosa*.

## 2. Results

### 2.1. Biopolymeric Nanoparticle Characterization

In the present study, different nanoparticle formulations (NS1, NS2, NC1, NC2, NC3, NC4, NC5, NC6 and NC7) were prepared to achieve higher encapsulation efficiency and greater system stability. Table 1 presents the results for pH, particle diameter (PD), zeta potential (ZP), recovery yields (Rec %) and encapsulation efficiency (EE %) of the prepared nanoparticles. The formulations exhibited a pH around 5. pH is an important parameter for evaluating the stability of nanoparticles in suspension and can serve as an indicator of polymer degradation or drug diffusion into the aqueous medium [33]. The particle diameter (PD) of the nanospheres prepared showed that all samples were within the nanometric range, with a mean diameter of <300 nm, and exhibited Rec % near 100%, demonstrating that the molecules remained stable during formulation and nanoparticle preparation. Zeta potential (ZP) results showed that both NSs and NCs exhibited a negative charge, with values ranging from –31.3 to –25.1 mV (Table 1). High zeta potential values, whether negative or positive (±30 mV), indicate the achievement of a stable suspension due to repulsion between particles, which prevents aggregation of nanoparticles [33]. EE % ranged from 79.3% to 98.0%. Peres et al., 2020 observed that EE % ranged from 86.0% to 99.0% for PCL nanoparticles containing essential oils from *Xylopia aromatica* leaves and fruits [34].

The amount of xanthyletin did not appear to affect the EE % of NCs, which remained consistently high (94.5 to 98.0%, Table 1). For NSs, however, an increase in xanthyletin content from 10 mg to 30 mg was associated with a noticeable reduction in EE %, from 91.9% to 79.3%. Isodecyl oleate was responsible for the most pronounced effect on particle diameter (PD). The presence of isodecyl oleate generated nanoparticles that were 50% larger than nanospheres for NC3, NC4 and NC5. A high xanthyletin level (30 mg) in NS formulations showed the presence of xanthyletin crystals, indicating that the maximum NS loading capacity had been reached under these conditions. Since the NSs had lower maximum load capacities than the NCs, it was decided to discontinue work with the NSs. After selecting the NCs as the working material, the isodecyl oleate/polymer ratio was investigated. An experiment was planned using isodecyl oleate/polymer ratios (NC3, NC4, NC5 and NC6). The goal of this second experiment was to reduce the percentage of non-encapsulated residual oil. After obtaining and centrifuging the formulations, the colloidal suspensions with higher isodecyl oleate/polymer ratios (NC1 and NC2) were found to be unstable and displayed free oil at the surface. This result suggested that the most efficient formulation for the xanthyletin-loaded nanoparticles should contain an isodecyl oleate/polymer ratio equal to or less than 0.6 g (NC5).

Colloidal suspensions with increasing amounts of xanthyletin were prepared to determine the maximum particle loading capacity. Colloidal suspensions with xanthyletin concentrations higher than 3300 µg·mL^−1^ (NC7) showed crystal formation, indicating that the maximum loading level for the formulation had been reached. The absence of crystals was also confirmed by the Rec % and EE % results, which averaged 96.5% and 98.0%, respectively, for NC5 (Table 1). The NC formulation containing xanthyletin (3000 µg·mL^−1^) with 150 mg PCL, 0.6 g isodecyl oleate, 50 mg Span^®^ 60 and 50 mg Tween^®^ 80 (NC5) was evaluated in terms of its morphology and subjected to stability studies (i.e., storage time, UV light-accelerated degradation and thermal degradation at 65 °C) and fungicidal bioassay.

### 2.2. Morphological Analysis

**Figure 1:**
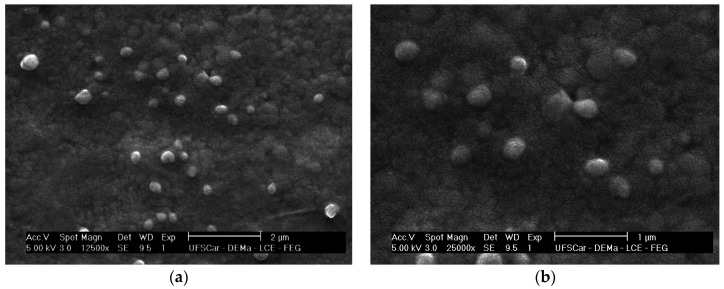
Scanning Electron Microscopy (SEM) images of PCL polymeric nanoparticles loaded with xanthyletin (NC5): (**a**) 12,500× magnification, with the scale bar width corresponding to 2 μm; (**b**) 25,000× magnification, with the scale bar width corresponding to 1 μm.

Figure 1 shows photomicrographs of the colloidal suspension of nanoparticles loaded with xanthyletin (NC5). The photomicrographs obtained did not show the formation of crystals at the concentration of xanthyletin used. Image analysis (Figure 1) confirmed the average particle size (PS) values and spherical morphology. It is important to emphasize that the average diameters of the empty nanoparticles did not differ significantly from the xanthyletin-loaded nanoparticles.

### 2.3. Stability Studies

**Figure 2:**
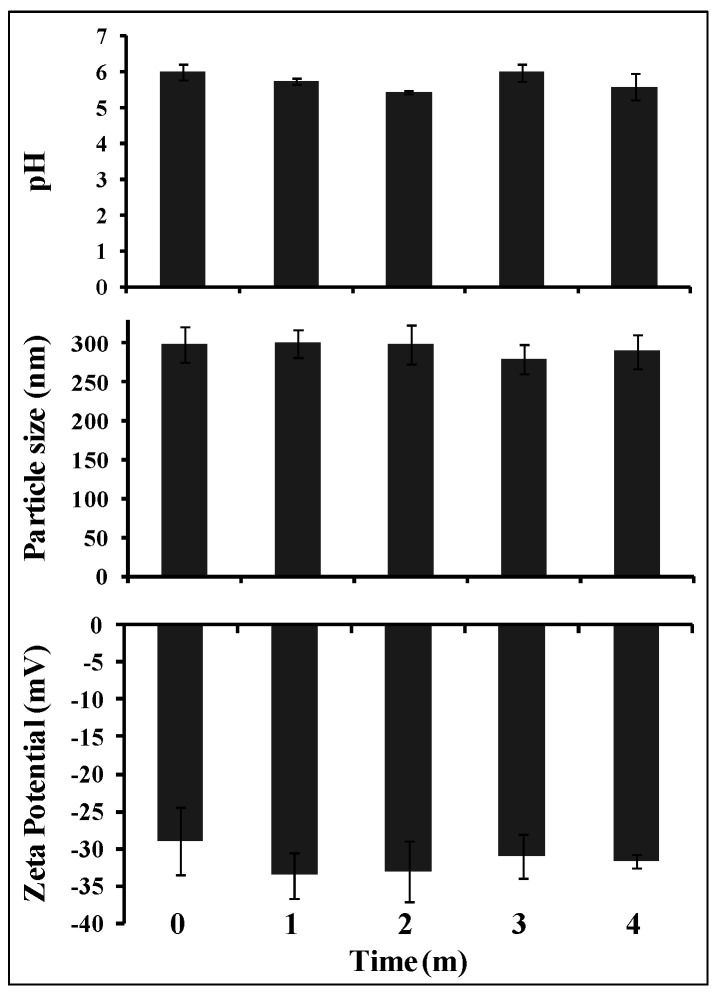
Average ZP, PS and pH variations of xanthyletin-loaded NC5 stored for 4 months at 4 °C. Each bar represents the average ± standard deviation values of three NC batches.

Colloidal nanoparticles tend to aggregate over time, especially when kept in aqueous dispersion form. Therefore, nanoparticle dimensions were evaluated during storage at 4 °C for four months to assess physical stability [35,36,37]. Under the present conditions, no significant variation in the average ZP value of xanthyletin-loaded NC5 was observed during the storage experiment (Figure 2). During polymer degradation, a reduction in pH was observed, as shown in Figure 2.

The visual appearance of the xanthyletin-loaded NCs remained unchanged. Neither opaque white suspensions nor surface oil was observed. At the end of the third month, small crystals were observed in the suspension. According to the literature, these crystals are associated with the formation of nanocrystals of the encapsulated substance during the preparation stage, which tend to grow and precipitate during storage [31,38].

### 2.4. In Vitro Release Studies

**Figure 3:**
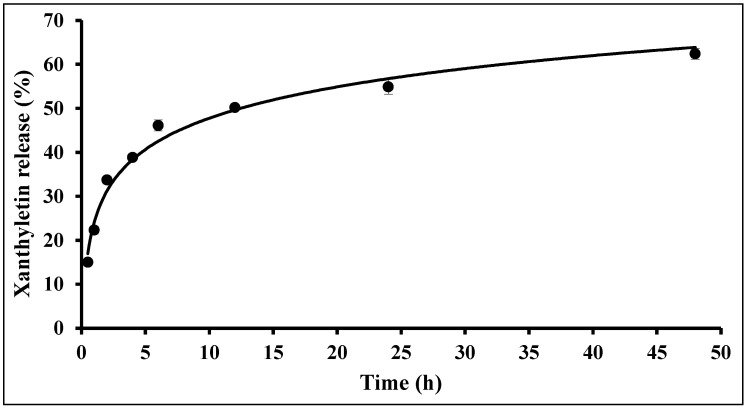
In vitro release profile of xanthyletin-loaded (NC5) colloidal dispersion (36 µg·mL^−1^) using the dialysis pocket diffusion technique. Each data point represents the average of three different NC batches.

The xanthyletin release profile from the PCL NC formulations developed in this study is shown in Figure 3. A biphasic release was observed, with an initial fast release, likely due to surface-adsorbed xanthyletin on the NCs (approximately 10 h), followed by a slower release during the second stage (after 10 h), most likely due to the diffusion of xanthyletin through the polymeric matrix combined with erosion.

The slow phase of release occurs only after the swelling process and/or degradation of the polymer matrix. The model described by Korsmeyer et al., 1983 [39], which is used for release mechanisms that are not fully established or when more than one phenomenon is involved, was applied after converting Equation (1) to its natural logarithm form. The empirically determined values for n and K, obtained between 0.5 and 48 h, were n = 0.2959 (n ≤ 45) and K = 0.370. This indicates a pure diffusion process, similar to the classical Fickian system, which is favored by the swelling of the polymer matrix [40].

### 2.5. UV Light-Accelerated Degradation

**Figure 4:**
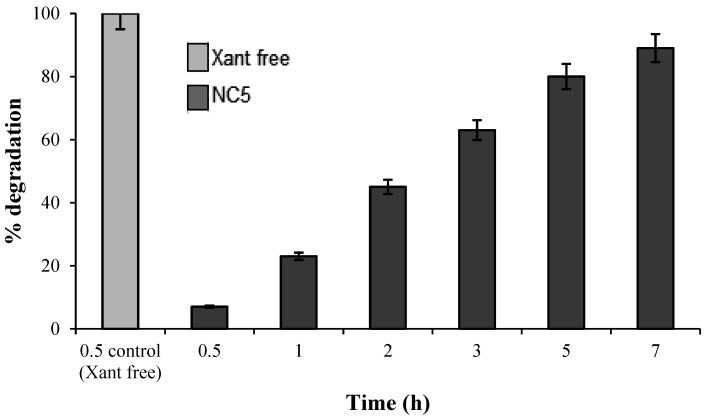
Degradation assay of free (Xant free) and nanoencapsulated (NC5) forms of xanthyletin exposed to UV light. Each bar represents the average value ± standard deviation (n = 3).

As shown in Figure 4, the in natura xanthyletin (without protection mechanisms) was completely degraded within thirty minutes of the start of the assay, 15.4 times faster compared to the encapsulated xanthyletin (NC5). These results demonstrated the protective efficiency provided by the nanoparticles on the stability of xanthyletin when exposed to UVA and UVB radiation.

These results corroborate the findings presented by Peres et al., 2020 [34], where the in natura essential oil of *X. aromatica* fruits, nanospheres and its control counterpart underwent 91%, 66% and 3% photodegradation, respectively, after 12 h of exposure.

### 2.6. Fungicidal Bioassay

**Figure 5:**
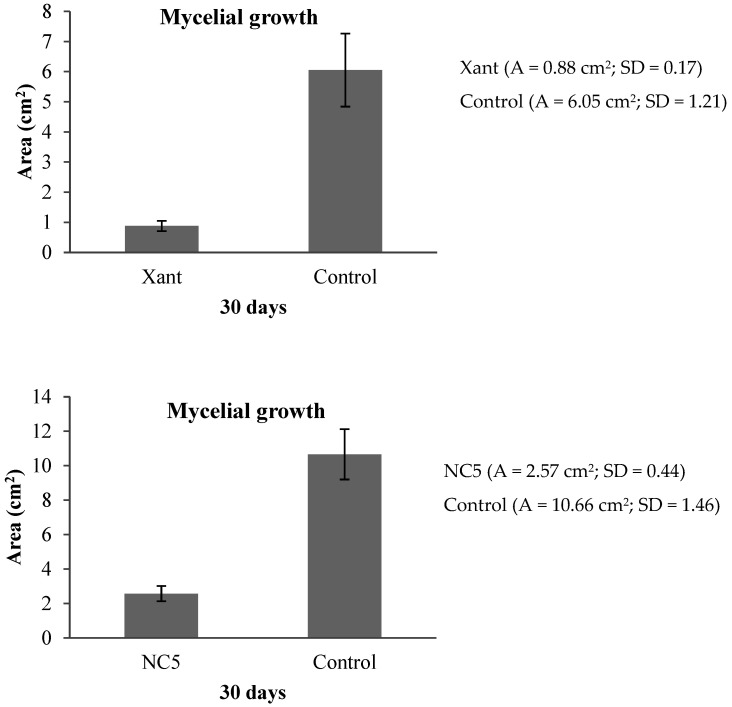
Effect of xanthyletin on the growth of the symbiotic fungus (*L. gongylophorus*). Free xanthyletin (Xant) and nanoencapsulated xanthyletin (NC5) were used to assess fungal growth after 30 days of inoculation. Values are expressed as the mean ± SD of five replicates.

**Figure 6:**
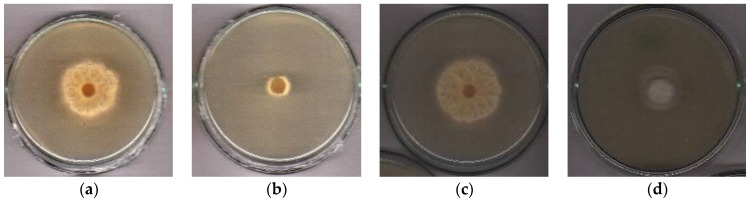
Symbiotic fungus images 30 days after inoculation: (**a**) control for free xanthyletin; (**b**) fungus treated with free xanthyletin; (**c**) control for nanoencapsulated xanthyletin (NC5); and (**d**) fungus treated with NC5.

The treatments with free xanthyletin and nanoencapsulated xanthyletin (NC5) exhibited 85% and 76% inhibitory activity on the symbiotic fungus (*L. gongylophorus*), respectively (Figure 5 and Figure 6). The activity of nanoencapsulated xanthyletin was approximately 10% lower than that of xanthyletin in solution.

## 3. Discussion

The present study describes, for the first time, the use of PCL in the preparation of nanoparticles containing the natural fungicidal xanthyletin. All formulations were macroscopically homogeneous, appearing as milky liquids with an opalescent whitish-blue color (Tyndall effect) due to nanoparticle formation. This characteristic aligns with previous literature reports [32,41], confirming the formation of nanoparticles [33]. A significant finding from this study was the substantial dispersion of xanthyletin in water. Xanthyletin’s low solubility in water can compromise its biological efficacy and diffusion in the environment. However, in this work, it was possible to prepare a colloidal suspension loaded with 3000 mg·L^−1^ of xanthyletin, which is approximately seventeen times higher than its concentration in aqueous solution.

The solvent displacement method using pre-formed PCL biopolymers was employed to prepare colloidal suspensions of xanthyletin-loaded NCs and NSs. This method offers several advantages, particularly its single-step execution, and allows for the use of low-toxicity solvents such as acetone and ethanol. Additionally, this method can be scaled for potential industrial production [42].

PCL was chosen as the polymer matrix due to its well-established biocompatibility, biodegradability and non-toxic nature. This aliphatic polyester has been extensively investigated, especially in drug delivery systems, due to its favorable degradation profile [43]. PCL degrades slowly via hydrolytic and enzymatic mechanisms, ultimately producing non-toxic byproducts [44]. Its biocompatibility and environmental degradability have also supported its broad application in both biomedical and agricultural fields, with minimal ecological risks [45].

The amount of PCL did not affect the loading capacity of the NCs and NSs. Similar results were reported by Peres et al., 2020, who developed PCL nanoparticles containing essential oils from *X. aromatica* leaves and fruits [34]. The current understanding of the polymer suggests that extracellular enzymes present in the soil can degrade the long polymer chains before microorganisms can incorporate the polymer [46]. Surfactants in nanometric systems provide stability to the nanoparticles. In this context, a high-HLB surfactant has been previously shown to prevent particle sedimentation and diffusion of the encapsulated active ingredient, while a low-HLB surfactant facilitated the formation of a small-sized, homogeneous nanoparticle population [38].

PCL polymeric nanoparticles loaded with xanthyletin (NC5) were observed. Similar results were previously reported by Christofoli et al., 2015, who obtained smooth, monodisperse nanospheres with diameters ranging from 400 to 500 nm using the PCL polymer as a matrix for encapsulating essential oils from *Zanthoxylum rhoifolium* leaves (Rutaceae) [47]. Additionally, Peres et al., 2020 also reported particle diameters smaller than 500 nm [34]. The release of the active ingredient from polymeric nanoparticles depends on several factors, including active ingredient desorption from the particle surfaces, diffusion through the NS matrix, diffusion through the NC polymeric wall, physicochemical or microbial erosion of the polymer matrix, or a combination of two or more of these processes [48]. The experiment was conducted under sink conditions, preventing any interference from the low hydrosolubility of the compound.

The control of leaf-cutting ants has traditionally relied on synthetic insecticides such as chlorpyrifos, deltamethrin, fipronil, permethrin and particularly sulfluramid [49]. However, sulfluramid was classified as a persistent organic pollutant under the Stockholm Convention, raising concerns about its environmental persistence and potential ecotoxicological effects [50]. As a result, alternative control strategies have been investigated, including approaches that target the mutualistic fungus *Leucoagaricus gongylophorus*, which plays a central role in colony maintenance and survival [18].

The selection of xanthyletin in the present study was based on previous findings demonstrating the antifungal potential of coumarins against *L. gongylophorus*. Godoy et al., 2005 [14] assessed eight coumarins isolated from four plant species and reported that, with the exception of clausarin, all compounds exhibited inhibitory activity at concentrations ranging from 64 to 80 μg·mL^−1^. Notably, xanthyletin achieved complete fungal inhibition at 25 μg·mL^−1^. More recently, Xiong et al., 2019 [15] reported the antifungal activity of xanthyletin against *Pyricularia oryzae*, reinforcing its broad-spectrum potential. To date, no studies have reported the antifungal efficacy of nanoencapsulated xanthyletin, highlighting the novelty and relevance of the present investigation.

The activity of nanoencapsulated xanthyletin was approximately 10% lower than that of free xanthyletin, which may be attributed to the controlled release kinetics of the encapsulated compound, as observed in previous studies on nanoencapsulated antifungals [51]. According to Miastkowska et al., 2020 [52], the encapsulation of antifungal compounds may initially reduce activity due to the controlled release of the active ingredient. However, this prolonged release maintains effective antifungal concentrations for extended periods, which is advantageous in agricultural practice. Additionally, for a bait to be successful against social insects, it is not desirable to induce high mortality among foraging workers. Instead, the bait should allow them to return to the nest, where it can impact the main target, the endosymbiotic fungi [53].

Although xanthyletin-loaded nanoparticles demonstrated efficacy against *Leucoagaricus gongylophorus*, their effects on non-target soil microorganisms, including beneficial fungi, remain unclear. Future studies should address the specificity and environmental safety of these nanoparticles to ensure minimal impact on soil microbial communities.

## 4. Materials and Methods

### 4.1. Plant Material

A collection of *Citrus sinensis* roots grafted onto *Citrus limonia* from the experimental farm of the Citriculture Experimental Station—Campinas Agronomic Institute in Cordeirópolis (São Paulo State, Brazil) was used in this study. The roots were dried in a convection oven at 40 °C for five days and then ground using a Wiley mill with a 500-µm mesh. The pulverized material (467.0 g) was macerated at room temperature with progressively polar organic solvents: *n*-hexane, dichloromethane and methanol (3.0 L each) for 72 h per solvent. The solvents were then evaporated using a rotary evaporator, yielding three extracts: hexane (5.73 g), dichloromethane (7.60 g) and methanol (17.64 g).

### 4.2. Purification and Identification of Xanthyletin

Xanthyletin was isolated from the dichloromethane extract of *Citrus sinensis* roots grafted on *Citrus limonia* using high-speed counter-current chromatography (HSCCC), following a methodology developed by our research group and detailed in a previous publication [54]. Structural identification was carried out based on UV and IR spectroscopy, as well as ^1^H and ^13^C NMR analyses.

### 4.3. Reagents and Standards

Poly-ε-(caprolactone) (PCL) with an average molecular weight of 65,000, sorbitan 60 monostearate (Span^®^ 60) and polysorbate 80 (Tween^®^ 80) were purchased from Sigma-Aldrich (St. Louis, MO, USA). Isodecyl oleate was acquired from Importadora Química Delaware Ltda. (Porto Alegre, Brazil). HPLC-grade solvents were sourced from J. T. Baker (Ecatepec, Mexico). Ultra-pure water was obtained via reverse osmosis using a Milli-Q system (Millipore, Bedford, MA, USA).

### 4.4. Nanoparticle Preparation

All nanoparticles, both NCs and NSs, were prepared using the pre-formed polymer nanoprecipitation technique (interfacial deposition/solvent displacement) as previously described [41,55,56]. In brief, an organic acetone (30 mL) phase containing the biopolymer (PCL), the active ingredient (xanthyletin), isodecyl oleate (used only for NC preparation) and Span^®^ 60 was prepared. This mixture was then slowly added to a constantly and magnetically stirred aqueous phase containing a high-hydrophilic-lipophilic balance (HLB) surfactant (Tween^®^ 80). The magnetic stirring was maintained for an additional 10 min to allow for stabilization. Subsequently, the organic solvent and part of the water were removed using a rotary evaporator at 45 °C, and the volume was adjusted accordingly. The varying ratios of xanthyletin (XANT) and isodecyl oleate were tested to optimize solubility, encapsulation efficiency and nanoparticle stability. Isodecyl oleate mass of 1000, 800, 700, 600 (NC5) and 500 mg were evaluated. Lower amounts were initially preferred due to cost and environmental considerations; higher ratios led to instability and free oil separation. The different formulations prepared are presented in Table 2.

### 4.5. Biopolymeric Nanoparticle Characterization

#### 4.5.1. Xanthyletin Quantification

The amount of xanthyletin in the nanoparticles was determined using a High-Performance Liquid Chromatograph (HPLC) system (Agilent Technologies (Sta. Clara, CA, USA, 1200 model) equipped with a G1311A quaternary pump, a G1322A degasser, a G1329A auto-injector, a G1316A column oven, and a G1314B UV detector. The system was coupled to a G1369A interface, and all chromatograms were recorded using Agilent EZChrom (version 3.3.2 SP2) software. A reverse-phase Phenyl Hexyl Phenomenex^®^ Luna column (250 × 4.6 mm i.d., 5 μm) was used, along with a Phenomenex C18 guard column (4 × 3 mm i.d., 5 μm). Chromatographic analyses were performed using an isocratic elution mode. The mobile phase consisted of a 60:40 (*v*/*v*) acetonitrile:water mixture, with a flow rate of 1.0 mL·min^−1^. The column temperature was maintained at 30 °C, and the injection volume was 20 µL. The UV-Vis detector was set to 263 nm for all experiments. All procedures for preparing standard samples and validating the analytical method were previously described [56].

#### 4.5.2. Determination of Total Xanthyletin Content

The total xanthyletin content was determined by dissolving aliquots of encapsulated xanthyletin nanoparticles in acetonitrile (1/500 dilution). The samples were analyzed in triplicate using HPLC, following the previously developed and validated analytical method [56].

#### 4.5.3. Determination of the Amount of Encapsulated Xanthyletin

The encapsulation efficiency (EE %) of xanthyletin in the nanoparticles was determined using the filtration-centrifugation technique. First, 0.5 mL of colloidal suspension was added to tubes containing 0.22-µm pore cellulose acetate filters (Costar^®^ Spin-X^®^, Corning Inc., Corning, NY, USA). The tubes were then centrifuged at 6800× *g* for 40 min using an Eppendorf^®^ centrifuge (Hamburg, HH, Germany, Model 5810R). The nanoparticles were retained by the filter membrane, while the aqueous phase (dispersion medium) passed through. Afterwards, 0.3 mL of the filtrate was collected, dried and resuspended in 0.2 mL of acetonitrile for HPLC analysis. The EE % was calculated by subtracting the amount of free xanthyletin in the filtrate from the total amount of xanthyletin, according to the following equation:

(1) $$\mathrm{EE}\%=\left[\left(C_{T}-C_{f}\right)/C_{T}\right]\times 100$$

where C_f_ represents the concentration of xanthyletin in the filtrate (µg·mL^−1^) and C_T_ represents the total concentration (µg·mL^−1^).

### 4.6. Particle Size and Zeta Potential Determination

The determination of particle pH, PS and ZP in suspension was performed immediately after the preparation of the nanoparticles. The pH values of the colloidal solution were determined using a potentiometer (B474 Micronal, São Paulo, Brazil). PS analyses were conducted using photon correlation spectroscopy (PCS). Nanoparticle PCS measurements were performed at room temperature at a fixed 90° angle. This technique provided data on both PS and the polydispersity index (PI)—distribution. The values of both PS and ZP were measured using a Zetatrac apparatus (Microtrac Inc., Montgomeryville, PA, USA), which was controlled with Microtrac Flex V.10.5.0 software (Microtrac Inc., USA). For each PCS and ZP (ζ, millivolts) measurement, 0.1 mL of each colloidal suspension was diluted to a final volume of 10.0 mL with ultra-pure water and 10 mmol·L^−1^ NaCl, respectively.

### 4.7. Morphological Analysis

To evaluate the homogeneity and morphology of the nanoparticles, droplets of the colloidal suspensions were deposited directly onto metallic supports. The samples were then coated with a thin gold layer and analyzed using scanning electron microscopy (SEM) (Philips XL 30 FEG, Amsterdam, NH, The Netherlands) at 10 kV, which magnified the samples by 50,000×.

### 4.8. Stability Studies

The colloidal suspensions underwent specific stability evaluation procedures. To assess shelf life stability, the pH, particle size (PS) and zeta potential (ZP) values of the suspensions were monitored at predetermined intervals over a 4-month period. The samples were stored in glass jars at 4 °C, protected from light. The UV light-accelerated degradation of the encapsulated materials was carried out in a UV-induced accelerated aging chamber (*l* = 60 cm, *h =* 40 cm and *w* = 60 cm). The chamber was equipped with four G15T8E USHIO lamps (15 W, *l* = 45 cm, *w* = 2.6 cm, USHIO, Tokyo, Japan), emitting irradiance of 3983 Mw m^−2^ and a chamber power output of 14.34 kJ·m^−2^·h^−1^. To optimize radiation utilization, the chamber walls were lined with mirrors, which are highly reflective and minimize luminosity losses. The system maintained a controlled temperature of 25.0 ± 2.0 °C through a thermostabilized air circulation system. Nanoparticle samples (1 mL per vial) were placed in the chamber and periodically monitored by HPLC to evaluate the stability of the encapsulated xanthyletin content during the UV light-accelerated degradation assays. The enhancement in photochemical stability due to nanoencapsulation was assessed. Control samples of xanthyletin in solution (non-encapsulated), both exposed and not exposed to light, were also used. The samples were analyzed over a period of 7 h, with results representing the average of three replicates.

### 4.9. Nanoparticle In Vitro Release Studies

In vitro release studies of colloidal suspension nanoparticles were performed using the dialysis pocket diffusion system originally proposed by Levy and Benita, 1990 [57]. Dialysis pockets (cellulose membrane tubing, 1.0 cm width, Sigma) containing 1.0 mL of colloidal suspension were sealed and placed in 200.0 mL of phosphate-buffered saline (PBS) at pH 7.4 under constant stirring. The PBS solution was prepared by dissolving the following salts per liter of water: 8.00 g NaCl, 0.20 g KCl, 1.44 g Na_2_HPO_4_·2H_2_O, and 0.24 g KH_2_PO_4_. The system was maintained at 35 °C. At specified time intervals, samples of the dispersion medium were collected, and the xanthyletin content was analyzed by HPLC. Release kinetics were determined by calculating the kinetic order parameter *n*, proposed by Korsmeyer et al., 1983 [39], using the empirical Equation (2):

(2) $$M_{t}/M_{\infty }=K\times t^{n}$$

where *M_t_*/*M_∞_* represents the release fraction at time interval *t*, *n* is the release exponent and *K* the release constant.

### 4.10. Fungicidal Bioassay

The experiments with the symbiotic fungus *L. gongylophorus* were conducted in the Bioassays Natural Products Laboratory at the Federal University of São Carlos (UFSCar). The maintenance media and methods for the bioassays were previously described by Bicalho et al., 2012 [58]. The fungus *L. gongylophorus* (Singer) Möller (syn. *Rozites gongylophorus*) was isolated from an *Atta sexdens rubropilosa* nest and maintained in the laboratory on a culture medium composed of malt extract (20 g·L^−^^1^), bacto-peptone (5 g·L^−^^1^), yeast extract (2 g·L^−^^1^) and agar (20 g·L^−^^1^). The samples submitted for assay with the symbiotic fungus were incorporated into the culture medium, followed by the addition of distilled water. Then, 10 mL of the culture medium with the sample or just the culture medium was added to each tube. The final xanthyletin concentration in both the bioassays with solutions and nanocapsules (colloidal suspension) was 100 µg·mL^−1^. The ingredients used in preparing the nanoparticles and empty nanocapsules (without xanthyletin) were also subjected to the fungicidal bioassay. All materials were autoclaved at 120 °C and 1.0 atm for 20 min, except for the nanocapsules. After sterilizing the materials, the culture media were poured into Petri dishes (80 × 15 mm) inside a laminar flow cabinet, which had been previously sterilized for 30 min with ultraviolet light. Once the culture medium solidified, each Petri dish was inoculated with an 8-mm agar disc, centrally placed, that had been previously colonized by the symbiotic fungus *L. gongylophorus*. The samples were prepared in quintuplicate, with an equivalent number of replicates assigned to the control group, which consisted of the culture medium and the symbiotic fungus. Following an incubation period of 30 days at 25 °C, the mycelial growth area of the symbiotic fungus in each sample was measured. The percentage of mycelial growth inhibition was determined by comparing the fungal growth area observed in the control plates with that of the plates containing the test samples. The control group was established as the reference, corresponding to 0% inhibition of the symbiotic fungus. Results are presented as mean values accompanied by standard deviation (±SD).

## 5. Conclusions

The nanoprecipitation method proved to be efficient for assembling xanthyletin-loaded NCs and NSs. Both NCs and NSs exhibited similar characteristics and high encapsulation efficiency (EE %) rates. However, the NCs demonstrated greater potential than the NSs, as the maximum loading capacity of xanthyletin in the NSs was lower than that of the corresponding NCs. The stability of the xanthyletin-loaded NCs was assessed in terms of storage time and UV radiation-accelerated degradation, and they were found to be more stable compared to xanthyletin in its natural form. In the in vitro release assay, a biphasic release pattern was observed, with a rapid initial release followed by slower release in the later stages. The encapsulation process proved to be an effective strategy for protecting the active ingredient from degradation induced by sunlight. Additionally, encapsulation enhances the solubility of xanthyletin in aqueous media, which could potentially reduce the frequency of applications, decrease the concentration of the applied doses, and thus minimize environmental impact. The results of the fungicidal bioassays demonstrated that both free xanthyletin and its nanoencapsulated form (NC5) exhibited antifungal activity against *Leucoagaricus gongylophorus*, with inhibition rates of 85% and 76%, respectively. Although the nanoencapsulated formulation presented approximately 10% lower activity compared to the free compound, it maintained effectiveness, suggesting its potential for use as a controlled-release system targeting the symbiotic fungus of leaf-cutting ants. These findings are preliminary and underscore the need for further investigation. Future studies, including field trials, will be essential to validate the in vitro results and assess the practical efficacy of the xanthyletin-loaded nanoparticle formulation in natural environments.

## Figures and Tables

**Table 1:** pH, particle diameter (PD), zeta potential (ZP), recovery yields (Rec %) and encapsulation efficiency (EE %) for various nanosphere (NSs) and nanocapsule (NCs) formulations.

Formulations	pH	PD (nm)	ZP (mV)	(Rec %)	(EE %)
NS1	5.91 ± 0.31	202 ± 1.58	−25.1 ± 0.09	97.2 ± 1.22	91.9 ± 0.83
NS2	5.92 ± 0.22	203 ± 0.98	−27.0 ± 0.11	99.2 ± 0.93	79.3 ± 0.72
NC3	5.87 ± 0.14	296 ± 1.22	−31.3 ± 0.07	98.4 ± 1.01	94.5 ± 0.48
NC4	5.94 ± 0.18	293 ± 0.99	−26.3 ± 0.13	98.9 ± 0.87	97.6 ± 0.79
NC5	5.33 ± 0.15	304 ± 1.17	−29.3 ± 0.07	96.5 ± 0.94	98.0 ± 0.97

**Table 2:** Formulation of nanospheres (NSs) and nanocapsules (NCs) prepared with xanthyletin.

Formulations	Xanthyletin (mg)	Isodecyl Oleate (mg)
NS1	10	0
NS2	30	0
NC1	10	1000
NC2	30	1000
NC3	30	800
NC4	30	700
NC5	30	600
NC6	30	500
NC7	33	600
**Fixed variables**		
Acetone	30.0 mL	
Span^®^ 60	50.0 mg	
Aqueous phase volume	60.0 mL	
Tween^®^ 80	50.0 mg	
PCL	150.0 mg	

## Data Availability

The original contributions presented in this study are included in the article. Further inquiries can be directed to the corresponding author(s).

## References

[1] Ejaz S., Akram W., Lim C.W., Lee J.J., Hussain I. (2004). Endocrine disrupting pesticides: A leading cause of cancer among rural people in Pakistan. Exp. Oncol..

[2] Gupta S., Gupta R., Sharma S. (2013). Impact of chemical- and bio-pesticides on bacterial diversity in rhizosphere of *Vigna radiata*. Ecotoxicology.

[3] Sparks T.C. (2013). Insecticide discovery: An evaluation and analysis. Pestic. Biochem. Physiol..

[4] Odukkathil G., Vasudevan N. (2013). Toxicity and bioremediation of pesticides in agricultural soil. Rev. Environ. Sci. Biotechnol..

[5] Kumar P., Kumar R., Thakur K., Mahajan D., Brar B., Sharma D., Kumar S., Sharma A.K. (2023). Impact of Pesticides Application on Aquatic Ecosystem and Biodiversity: A Review. Biol. Bull..

[6] Qamar W., Shahid M.U., Irfan M., Abbas R.Z., Faraz A., Hussain R., Alvi M.A. (2023). Thiamethoxam Toxicity: A Review in One-Health Perspective. Kafkas Univ. Vet. Fak. Derg..

[7] Pinto A.C., Silva D.H.S., Bolzani V.S., Lopes N.P., Epifanio R.A. (2002). Produtos naturais: Atualidade, desafios e perspectivas. Quim. Nova.

[8] Castâno-Quintana K., Montoya-Lerma J., Giraldo-Echeverri C. (2013). Toxicity of foliage extracts of *Tithonia diversifolia* (*Asteraceae*) on *Atta cephalotes* (Hymenoptera: Myrmicinae) workers. Ind. Crops Prod..

[9] Sparks T.C., Bryant R.J. (2022). Innovation in insecticide discovery: Approaches to the discovery of new classes of insecticides. Pest Manag. Sci..

[10] Abdelgaleil S.M.A., Gad H.A., Ramadan G.R.M., El-Bakry A.M., ElSabrout A.M. (2023). Monoterpenes for Management of Field Crop Insect Pests. J. Agric. Sci. Technol..

[11] Zhang P., Qin D., Chen J., Zhang Z. (2020). Plants in the Genus *Tephrosia*: Valuable Resources for Botanical Insecticides. Insects.

[12] Kilani-Morakchi S., Morakchi-Goudjil H., Sifi K. (2021). Azadirachtin-Based Insecticide: Overview, Risk Assessments, and Future Directions. Front. Agron..

[13] Araújo M.F., Castanheira E.M.S., Sousa S.F. (2023). The Buzz on Insecticides: A Review of Uses, Molecular Structures, Targets, Adverse Effects, and Alternatives. Molecules.

[14] Godoy M.F.P., Victor S.R., Bellini A.M., Guerreiro G., Rocha W.C., Bueno O.C., Hebling M.J.A., Bacci M.C., da Silva M.F.G.F., Vieira P.C. (2005). Inhibition of the symbiotic fungus of leaf-cutting ants by coumarins. J. Braz. Chem. Soc..

[15] Xiong Y., Huang G., Yao Z., Zhao C., Zhu X., Wu Q., Zhou X., Li J. (2019). Screening Effective Antifungal Substances from the Bark and Leaves of *Zanthoxylum avicennae* by the Bioactivity-Guided Isolation Method. Molecules.

[16] Boulogne I., Ozier-Lafontaine H., Germosen-Robineau L., Desfontaines L., Loranger-Merciris G. (2012). *Acromyrmex octospinosus* (Hymenoptera: Formicidae) management: Effects of TRAMILs fungicidal plant extracts. J. Econ. Entomol..

[17] Motta A.C.Q., Avelino D.S., Marinho C.G.S., Fadini M.A.M., Melo J.O.F. (2022). *Leucoagaricus gongylophorus* provides protection for *Atta sexdens* against plant extracts. Cienc. Florest..

[18] Araújo S., Seibert J., Ruani A., de la Cruz R.A., Cruz A., Pereira A., Doraí Zandonai D., Forim M., Silva M.F., Bueno O. (2022). The Symbiotic Fungus *Leucoagaricus gongylophorus* (Möller) Singer (*Agaricales*, *Agaricaceae*) as a Target Organism to Control Leaf-Cutting Ants. Insects.

[19] Zanuncio J.C., Lopes E.T., Leite H.G., Zanetti R., Sediyama C.S., Fialho M.C.Q. (2004). Sampling methods for monitoring the number and area of colonies of leaf-cutting ants (Hymenoptera: Formicidae) in Eucalyptus plantations in Brazil. Sociobiology.

[20] Nickele M.A., Reis Filho W., Oliveira E.B., Iede E.T., Caldato N., Strapasson P. (2012). Leaf-cutting ant attack in initial pine plantations and growth of defoliated plants. Pesqui. Agropecuária Bras..

[21] Buteler M., Alma A.M., Herrera M.L., Gorosito N.B., Fernández P.C. (2019). Novel organic repell ent for leaf-cutting ants: Tea tree oil and its potential use as a management tool. Int. J. Pest Manag..

[22] Justi Junior J., Imenes S.L., Bergmann E.L., Campos-Farinha A.E.C., Zorzenon F.J. (1996). Formigas cortadeiras. Bol. Técn. Inst. Biol..

[23] Liu Y., Tong Z., Prud’homme R.K. (2008). Stabilized polymeric nanoparticles for controlled and efficient release of bifenthrin. Pest Manag. Sci..

[24] Paula H.C.B., Sombra F.M., Abreu F.O.M.S., De Paula R.C.M. (2010). *Lippia sidoides* essential oil encapsulation by angico gum/chitosan nanoparticles. J. Braz. Chem. Soc..

[25] Yang F.L., Li X.G., Zhu F., Lei C.L. (2009). Structural characterization of nanoparticles loaded with garlic essential oil and their insecticidal activity against *Tribolium castaneum* (Herbst) (Coleoptera: Tenebrionidae). J. Agric. Food Chem..

[26] Beyki M., Zhaveh S., Khalili S.T., Rahmani-Cherati T., Abollahi A., Bayat M., Tabatabaei M., Mohsenifar A. (2014). Encapsulation of *Mentha piperita* essential oils in chitosan–cinnamic acid nanogel with enhanced antimicrobial activity against *Aspergillus flavus*. Ind. Crops Prod..

[27] Forim M.R., Costa E.S., da Silva M.F.G.F., Fernandes J.B., Mondego J.M., Boiça A.L. (2013). Development of a new method to prepare nano-/microparticles loaded with extracts of *Azadirachta indica*, their characterization and use in controlling *Plutella xylostella*. J. Agric. Food Chem..

[28] Buteler M., Garcia G.L., Stadler T. (2018). Potential of nanostructured alumina for leaf-cutting ants *Acromyrmex lobicornis* (Hymenoptera: Formicidae) management. Austral Entomol..

[29] Decool G., Kfoury M., Paitel L., Sardo A., Fourmentin S. (2023). Cyclodextrins as molecular carriers for biopesticides: A review. Environ. Chem. Lett..

[30] Zielinska A., Carreiró F., Oliveira A.M., Neves A., Pires B., Venkatesh D.N., Durazzo A., Lucarini M., Eder P., Silva A.M. (2020). Polymeric Nanoparticles: Production, Characterization, Toxicology and Ecotoxicology. Molecules.

[31] Schaffazick S.R., Pohlmann A.R., Freitas L.D., Guterres S.S. (2002). Caracterização e estudo de estabilidade de suspensões de nanocápsulas e de nanoesferas poliméricas contendo diclofenaco. Acta Farm. Bonaer..

[32] Schaffazick S.R., Guterres S.S.U., Freitas L.D., Pohlmann A.R. (2003). Physicochemical characterization and stability of the polymeric nanoparticle systems for drug administration. Quim. Nova.

[33] Nascimento T.G., Silva P.F., Azevedo L.F., Rocha L.G., Moraes Porto I.C.C.M., Moura T.F.A.L., Basílio-Júnior I.D., Grillo L.A.M., Dornelas C.B., Fonseca E.J.S. (2016). Polymeric Nanoparticles of Brazilian Red Propolis Extract: Preparation, Characterization, Antioxidant and Leishmanicidal Activity. Nanoscale Res. Lett..

[34] Peres M.C., Costa G.C.S., Reis L.E.L., Silva L.D., Peixoto M.F., Alves C.C.F., Forim M.R., Quintela E.D., Araújo W.L., Cazal C.M. (2020). In natura and nanoencapsulated essential oils from *Xylopia aromatica* reduce oviposition of *Bemisia tabaci* in *Phaseolus vulgaris*. J. Pest Sci..

[35] Bala I., Hariharan S., Kumar M. (2004). PLGA nanoparticles in drug delivery: The state of the art. Crit. Rev. Ther. Drug Carr. Syst..

[36] Bodmeier R.D., Mainceint O., Lieberman H.A., Rieger M.M., Banker G.S. (1998). Polymer dispersions as drug carriers. Pharmaceutical Dosage Forms: Disperse Systems.

[37] Magenheim B., Benita S. (1991). Nanoparticle characterization: A comprehensive physicochemical approach. Pharma Sci..

[38] Guterres S.S., Fessi H., Barratt G., Devissaguet J.P., Puisieux F. (1995). Poly-(DL-lactide) nanocapsules containing diclofenac, 1. Formulation and stability study. Int. J. Pharm..

[39] Korsmeyer R.W., Gurny R., Doelker E., Buri P., Peppas N.A. (1983). Mechanism of solute release from porous hydrophilic polymers. Int. J. Pharm..

[40] Li Z.Z., Xu S.A., Wen L.X., Liu F., Liu A.Q., Wang Q., Sun H.Y., Yu W., Chen J.F. (2006). Controlled release of avermectin from porous hollow silica nanoparticles: Influence of shell thickness on loading efficiency, UV-shielding property and release. J. Control. Release.

[41] Fessi H., Puisieux F., Devissaguet J.P., Ammoury N., Benita S. (1989). Nanocapsule formation by interfacial polymer deposition following solvent displacement. Int. J. Pharm..

[42] Galindo-Rodriguez S.A., Puel F., Briancon S., Allemann E., Doelker E., Fessi H. (2005). Comparative scale-up of three methods for producing ibuprofen-loaded nanoparticles. Eur. J. Pharm. Sci..

[43] Dash T.K., Konkimalla V.B. (2011). Polymeric materials for drug delivery systems. J. Control. Release.

[44] Woodruff M.A., Hutmacher D.W. (2010). The Return of a Forgotten Polymer-Polycaprolactone in the 21st Century. Prog. Polym. Sci..

[45] Choi J.-H., Woo J.-J., Kim I. (2023). Sustainable Polycaprolactone Polyol-Based Thermoplastic Poly(ester ester) Elastomers Showing Superior Mechanical Properties and Biodegradability. Polymers.

[46] Rosa D.S., Penteado D.F., Calil M.R. (2000). Thermal properties and biodegradability of PCL and PHB submitted in fungi pool. Rev. Cienc. Tecnol..

[47] Christofoli M., Costa E.C.C., Bicalho K.U., Domingues V.C., Peixoto M.F. (2015). Insecticidal effect of nanoencapsulated essential oils from *Zanthoxylum rhoifolium* (Rutaceae) in *Bemisia tabaci* populations. Ind. Crop Prod..

[48] Soppimath K.S., Aminabhavi T.M., Kulkarni A.R., Rudzinski W.E. (2001). Biodegradable polymeric nanoparticles as drug delivery devices. J. Control. Release.

[49] Vinha G.L., Alcántara-de la Cruz R., Della Lucia T.M.C., Wilcken C.F., da Silva E.D., Lemes P.G., Zanuncio J.C. (2020). Leaf-cutting ants in commercial forest plantations of Brazil: Biological aspects and control methods. South. J. Sci..

[50] Torres F.B.M., Guida Y., Weber R., Torres J.P.M. (2022). Brazilian overview of per- and polyfluoroalkyl substances listed as persistent organic pollutants in the Stockholm Convention. Chemosphere.

[51] Yammine J., Chihib N.-E., Gharsallaoui A., Dumas E., Ismail A., Karam L. (2022). Essential oils and their active components applied as: Free, encapsulated and in hurdle technology to fight microbial contaminations. Heliyon.

[52] Miastkowska M., Michalczyk A., Figacz K., Sikora E. (2020). Nanoformulations as a modern form of biofungicide. J. Environ. Health Sci. Eng..

[53] Della Lucia T.M., Gandra L.C., Guedes R.N. (2014). Managing leaf-cutting ants: Peculiarities, trends and challenges. Pest Manag. Sci..

[54] Cazal C.M., Domingues V.C., Batalhao J.R., Bueno O.C., Rodrigues Filho E., da Silva M.F.G.F., Vieira P.C., Fernandes J.B. (2009). Isolation of xanthyletin, an inhibitor of ants’ symbiotic fungus, by high-speed counter-current chromatography. J. Chromatogr. A.

[55] Fessi H., Puisieux F., Devissaguet J.P. (1988). Procédé de Préparation de Systémes Colloidaux Dispersibles d’une Substance Sous Forme de Nanoparticules.

[56] Cazal C.M., Forim M.R., da Silva M.F.G.F., Vieira P.C., Fernandes J.B. (2014). Development and validation of a RP-HPLC method to determine the xanthyletin content in biodegradable polymeric nanoparticles. Quim. Nova.

[57] Levy M.Y., Benita S. (1990). Drug release from submicronized o/w emulsion: A new in vitro kinetic evaluation model. Int. J. Pharm..

[58] Bicalho K.U., Terezan A.P., Martins D.C., Freitas T.G., Fernandes J.B., da Silva M.F.G.F., Vieira P.C., Pagnocca F.C., Bueno O.C. (2011). Evaluation of the toxicity of *Virola sebifera* crude extracts, fractions and isolated compounds on the nest of leaf-cutting ants. Psyche.

